# The Analysis of Live-Attenuated *Piscirickettsia salmonis* Vaccine Reveals the Short-Term Upregulation of Innate and Adaptive Immune Genes in Atlantic Salmon (*Salmo salar*): An In Situ Open-Sea Cages Study

**DOI:** 10.3390/microorganisms9040703

**Published:** 2021-03-29

**Authors:** Deborah Vargas, Eva Vallejos-Vidal, Sebastián Reyes-Cerpa, Aarón Oyarzún-Arrau, Claudio Acuña-Castillo, Mónica Imarai, Felipe E. Reyes-López, Ana María Sandino

**Affiliations:** 1 Consorcio Tecnológico de Sanidad Acuícola, Ictio Biotechnologies S.A., 7500652 Santiago, Chile; deborah.vargas@usach.cl (D.V.); aaron.oyarzuna@usach.cl (A.O.-A.); monica.imarai@usach.cl (M.I.); 2 Departamento de Biología, Facultad de Química y Biología, Universidad de Santiago de Chile, 9170002 Santiago, Chile; claudio.acuna@usach.cl; 3 Centro de Biotecnología Acuícola, Facultad de Química y Biología, Universidad de Santiago de Chile, 9170002 Santiago, Chile; eva.vallejosv@usach.cl; 4 Centro de Genómica y Bioinformática, Facultad de Ciencias, Universidad Mayor, 8580745 Santiago, Chile; sebastian.reyes@umayor.cl; 5 Escuela de Biotecnología, Facultad de Ciencias, Universidad Mayor, 8580745 Santiago, Chile; 6 Department of Cell Biology, Physiology and Immunology, Universitat Autònoma de Barcelona, 08193 Bellaterra, Spain; 7 Facultad de Medicina Veterinaria y Agronomía, Universidad de Las Américas, 7500975 Providencia, Chile

**Keywords:** *Piscirickettsia salmonis*, Salmonid Rickettsial Septicemia (SRS), attenuated live vaccine, cellular immunity, interferon-mediated immune response, open sea cage farming

## Abstract

*Piscirickettsia salmonis*, the etiological agent of the Salmon Rickettsial Septicemia (SRS), is one the most serious health problems for the Chilean salmon industry. Typical antimicrobial strategies used against *P. salmonis* include antibiotics and vaccines, but these applications have largely failed. A few years ago, the first attenuated-live vaccine against SRS (ALPHA JECT LiVac^®^ SRS vaccine) was released to the market. However, there is no data about the agents involved in the activation of the immune response induced under field conditions. Therefore, in this study we evaluated the expression profile of a set of gene markers related to innate and adaptive immunity in the context of a cellular response in Atlantic salmon (*Salmo salar*) reared under productive farm conditions and immunized with a live-attenuated vaccine against *P. salmonis*. We analyzed the expression at zero, 5-, 15- and 45-days post-vaccination (dpv). Our results reveal that the administration of the attenuated live SRS LiVac vaccine induces a short-term upregulation of the cellular-mediated immune response at 5 dpv modulated by the upregulation of *ifnα*, *ifnγ*, and the *cd4* and *cd8α* T cell surface markers. In addition, we also registered the upregulation of *il-10* and *tgfβ*. Altogether, the results suggest that a balanced activation of the immune response took place only at early times post-vaccination (5 dpv). The scope of this short-term upregulation of the cellular-mediated immune response against a natural outbreak in fish subjected to productive farm conditions deserves further research.

## 1. Introduction

In 2018, about 88% of the 179 million tons of total fish production was utilized for direct human consumption, a share that has increased significantly in recent decades. The contribution of world aquaculture to world fish production has constantly increased, reaching 46.0% in 2016–2018, valued at USD 250 billion. China is by far the main world exporter of fish and fish products, followed by Norway, Vietnam, India and Chile. In Chile, aquaculture production of Atlantic salmon (*Salmo salar*), coho salmon (*Oncorhynchus kisutch*) and rainbow trout (*Oncorhynchus mykiss*) has grown strongly, thus consolidating its position as second worldwide salmon producer just behind Norway. Chile has seen sustained export revenue growth on the back of strong global demand for salmonids throughout the Americas, Europe and Asia and an increase in prices, reaching export revenues of USD 6.6 billion [1].

Under intensive culture conditions, aquatic animal disease is one of the most serious constraints to the expansion and development of sustainable aquaculture [2]. Fish may be exposed to several environmental and husbandry related stimuli that may have a potential noxious or stressful effect. All these factors have negative impacts on fish welfare, overall performance, and protective immune response, increasing the susceptibility to disease [3]. Farmed fish are continually exposed to pathogens, including bacteria, viruses, and parasites, which may produce outbreaks and mortality [4,5]. Prevention of diseases is an advisable practice in aquaculture, which is based on improved husbandry practices, movement restrictions, genetically resistant-disease stock, dietary supplements, non-specific immunostimulants, vaccine, probiotics, prebiotics, medicinal plant products, water disinfection and antimicrobial compounds as the best approaches in controlling infectious diseases of fish [4,6]. However, effective vaccines are probably the most important factors for the growth and success of intensive salmonid farming systems. The development of a sustainable aquaculture industry depends on the development and implementation of vaccines and vaccination regimes that makes the disease situation predictable and manageable under intensive production [7].

In Chile, the National Fisheries Service (SERNAPESCA, *Servicio Nacional de Pesca*) identified along their coasts the presence of *Piscirickettsia salmonis*, the etiological agent of Salmonid Rickettsial Septicaemia (SRS). *P. salmonis* is probably the most serious health problem for the Chilean salmon industry [8], because of its highly aggressive nature, recurrent outbreaks, and widespread transmission among other cultivated salmonid species [9,10,11,12]. In 2018, mortalities associated with *P. salmonis* represented 54.7% and 83.3% of the total mortalities attributed to infectious causes in Atlantic salmon and rainbow trout, respectively [9]. The control and prophylactic strategies against *P. salmonis* have relied on antibiotics and vaccines to date; however, both are inadequate [13]. Antibiotics have been used inappropriately to control outbreaks of infection, an excessive use of mainly florfenicol and oxytetracycline generating concern about public health [14]. Moreover, infected salmonids respond poorly to these treatments, likely because of the intracellular characteristics of the infective cycle of *P. salmonis* and the insufficient concentrations of antibiotics that reach the intracellular niche to eliminate the bacterium [13]. This situation is further complicated by the lack of effective vaccines against *P. salmonis* because prophylactic vaccines do not provide acceptable levels of protection [4,13].

Today, several commercial vaccines are available against *P. salmonis*. Most of them are based on bacterin, defined as a preparation of killed bacteria based on heat-inactivation or formalin treatment. Also available are multivalent bacterin, which contains antigens from *V. ordalii* (anguillarum), *A. salmonicida*, IPNV, ISAV and/or *C. rogercresseyi*. Moreover, there vaccines are available with recombinant proteins, such as AQUAVAC^®^ SARISTIN 2, which contain VP2 protein from IPNV and ORF1-90 kDa from *P. salmonis*, and BIRNAGEN FORTE 2, which contains recombinant proteins HSP70, HP60 and FLG G2 from *P. salmonis* and inactivated IPNV. Finally, ALPHA JECT LiVac^®^ SRS is the available live-attenuated vaccine currently in use in the Chilean salmonid aquaculture industry [13,15].

Protection induced by vaccines against *P. salmonis* administered under controlled conditions in the laboratory is well documented. However, protection under field conditions appears to be contradictory, with a lack of information regarding immune response [13,16,17]. The experience is that vaccines against *P. salmonis* confer good short-term protection against disease and mortality, but are inefficient in conferring long-term protection [17].

The immune system in teleost fish has similarities to the mammalian immune system [18,19]. The innate immunity of fish is composed of physical, humoral and cellular defense barriers. The physical barriers act as the first line of defense (skin, gills and mucous membranes). The innate humoral immunity emerges from the enzymes, complement proteins and opsonizing antibodies. Finally, the cellular barrier is composed by leukocytes (macrophages, neutrophils, natural killer (NK) and eosinophils) with morphological and functional similarities to mammals’ leukocytes [19]. Nevertheless, an adaptive immunity has also been described in teleost, made of T and B lymphocytes, together with a cytokine network and a production of specific antibodies. However, the adaptive immune response has been described as slower than that of mammals, with a more limited repertoire, where specific antibodies are not detected until three or four weeks after immunization, thus making it a less efficient response than the one described for mammals [13,20].

Vaccination strategies using injectable and oral vaccines have been shown to induce an immune response mediated by antibodies [21]. However, the intracellular nature of the infective cycle of *P. salmonis* [13] suggests that the activation of a cellular-mediated mechanism should take place for a successful host protection. Importantly, there are no data about the mechanisms of induction for conferring the short-term protection observed in fish farming. For this reason, in this study we explored the gene expression profile of molecules associated with the cellular-mediated immune response in Atlantic salmon immunized with a live-attenuated vaccine against *P. salmonis*, evaluated at zero, 5-, 15- and 45-days post-vaccination (dpv). The analysis included the expression profile of innate (complement component 3 (*c3*); interferon alpha (*ifnα*)) and adaptive immune response gene markers including ifn gamma (*ifnγ*) (associated to T helper 1 (Th1) cells response), *il-10* and *tgf-β* (for regulatory response), and *cd4* and *cd8α* (T cell surface markers). We focus on the differential expression pattern between vaccinated and non-vaccinated fish.

## 2. Materials and Methods

### 2.1. Fish

The study included a total of 163,590 Atlantic salmon (*Salmo salar*) (body weight (BW) mean 904.8 g) reared on the Chilean southern coast (Hornopirén; Región de Los Lagos). The assay was conducted in a commercial aquaculture farm under an open-sea cages productive regime. The experiment lasted for 45 days from October (11.4 °C) to December 2017 (12.8 °C). Water parameters were monitored daily according to the Chilean sanitary regulations in force.

### 2.2. Vaccination Trial and Sampling

At the moment of performing the experiment, fish were already distributed in their cages for commercial purposes following routine productive practices. The trial included two sea cages: one cage for the vaccinated group (*n* = 80,189 fish; BW mean 1045 g; density = 4.4 Kg/m^3^); and a different cage for the non-vaccinated fish (*n* = 83,401 fish; BW mean 764.5 g; density = 3.4 Kg/m^3^). All non-vaccinated and vaccinated fish included in the study were weighed at zero (1143 ± 286 g) and at 5 dpv (962 ± 153 g; 1110 ± 192 g), 15 dpv (937 ± 126 g; 1167 ± 272 g), and 45 dpv (1443 ± 323 g; 1651 ± 341 g), respectively. Fish were starved 24 h before vaccination. The ALPHA JECT LiVac^®^ SRS (AL 20,542 strain, 1.9 × 10^5^ –4.9 × 10^6^ TCID50; PharmaQ AS, Overhalla, Norway) was used as live-attenuated *Piscirickettsia salmonis* vaccine source. Fish were fasted for 24 h prior to vaccination. The intraperitoneal vaccination trial (100 µL per fish) was conducted after sedation with MS222 (50 mg/L) and following the manufacturer’s recommendations. In this study, the gene expression profile was evaluated in the head kidney because of the evidence of *P. salmonis* infection, survival and replication in head kidney macrophages [22,23]. Head kidney was sampled from both vaccinated and non-vaccinated fish at zero, 5-, 15-, and 45-days post-vaccination (dpv) (*n* = 15 fish per treatment and sampling time-point). The cumulative survival was monitored daily (99.91% survival for vaccinated fish: 99.47% for non-vaccinated fish). Before sampling, fish were euthanized by over anesthetization in MS222 (200 mg/L). Samples were preserved in RNAlater (Invitrogen, Thermo Fisher Scientific, Vantaa, Finland), incubated overnight (4 °C), and stored immediately afterwards at −80 °C until sample processing for total RNA extraction.

### 2.3. RNA Extraction and cDNA Synthesis

Total RNA from head kidney samples (*n* = 15 fish per treatment and sampling time-point) was obtained using TRIsure (Bioline, London, UK). The RNA pellet was resuspended in 100 µL nuclease-free water. The total RNA was quantified spectrophotometrically with the Infinitive 200Pro (TECAN Austria GmbH, Grödig, Austria). The RNA integrity was also inspected by 1% agarose gel. Only those samples with 260:280 ratio greater than 1.7 and integrity with no apparent sign of degradation were considered. Total RNA (2.0 µg) samples were treated with the RQ1 RNase-free DNase (Promega, Madison, WI, USA) before performing cDNA synthesis using reverse transcriptase M-MLV (Promega) and Oligo dT (Promega) following the manufacturer’s recommendations. Once reactions were completed, the cDNA samples were stored until use at −20 °C.

### 2.4. Gene Expression Analysis

The head kidney from vaccinated and non-vaccinated fish were analyzed by RT-qPCR. For this purpose, the ImmuneTrack kit (Ictio Biotechnologies SA, Santiago, Chile) was used for assessing the innate and adaptive immune genes according to the manufacturer’s instructions. Thus, the expression profile of innate (*c3*; *ifnα*) and adaptive immune response gene markers including *ifnγ*, *il-10*, *tgf-β*, *cd4,* and *cd8α* was evaluated. We tested the expression of *β-Actin* (ImmuneTrack kit) and *18S* (primer sequences described in [24]) in order to elucidate the better reference gene for the study. Thus, the expression of elongation factor 1 alpha (*ef1α*) was included as reference gene based on the low variation between all the samples included in the analysis. Importantly, the use of ef1α was suggested as reference gene in RT-qPCR assays for studying the effect of *P. salmonis* on the host immune response [25].

Quantitative PCR reactions were performed with 5.0 μL 2X buffer reaction SensiFAST SYBR No-ROX Kit (Bioline), 0.25 μL forward and reverse primers (10 µM concentration stock each), 3.5 μL of miliQ H_2_O, and 1.0 μL from each of all the cDNA stock samples. The thermal conditions are detailed on Table 1. At the end of the last qPCR reaction cycle, a temperature ramping step from 65 to 95 °C was included to produce the melting curves in order to verify the amplification of a unique single product for all the samples included in the gene expression analysis. All the reactions were performed in duplicate using the Thermo Scientific PikoReal Real-Time PCR System (Thermo Fisher, Vantaa, Finland). Quantification was done according to the Livak method [26]. The normalized relative expression (NRE) value for each condition (control and vaccinated fish) was calculated using the time zero (calibrator) and normalized to the *ef1a* (reference gene) expression. The results were expressed as mean expression values obtained at zero, 5, 15, and 45 days after vaccination (*n* = 15 fish per group, experimental condition and time-point assessed).

The presence of the most infectious/persistent pathogens affecting the Chilean salmon industry including virus (IPNV, ISAV, PRV) and bacteria (*P. salmonis*, *R. salmoninarum*, and *F. psychrophilum*) was checked by qRT-PCR using Kit Plus (Ictio Biotechnologies SA, Chile). No amplification was registered for any of the pathogens assessed in the samples studied.

### 2.5. Statistical Analysis

All statistical analysis and graphs were conducted using Graph Pad Prism V.6.1. Software (GraphPad Software, San Diego, CA, USA). Two-way ANOVA test was used to determine differences in gene expression between control and vaccinated groups (all data were checked for normality and homoscedasticity). To find differences for each time between control and vaccinated groups, a post-hoc Sidak’s multiple comparisons test was conducted (α = 0.05). Gene expression results are expressed as the mean ±SD (standard deviation). Statistical differences were indicated when *p* value ≤ 0.05. To calculate the percentage of survival for control and vaccinated groups the product limit (Kaplan-Meier) method was used.

## 3. Results

The short-term effect of the ALPHA JECT LiVac^®^ SRS vaccine upon the modulation of immune markers was evaluated at zero, 5-, 15- and 45-days post-vaccination (dpv) in an Atlantic salmon farm located in the south of Chile and subjected to an open sea productive system. We assessed the expression of genes associated with the immune response using the ImmunoTrack I (for Innate-related genes) and the ImmunoTrack A (for Adaptive-related genes) commercial kits. Thus, the strategy included the analysis of innate (complement component 3 (*c3*); interferon alpha (*ifnα*)) and adaptive genes related to T helper (Th) response (ifn gamma (*ifnγ*) for Th1; interleukin (*il*)-10 for Th2), regulatory mechanisms (*tgf-β1*; *il-10*), and immune cell surface markers (*cd4*; *cd8α*).

**Figure 1:**
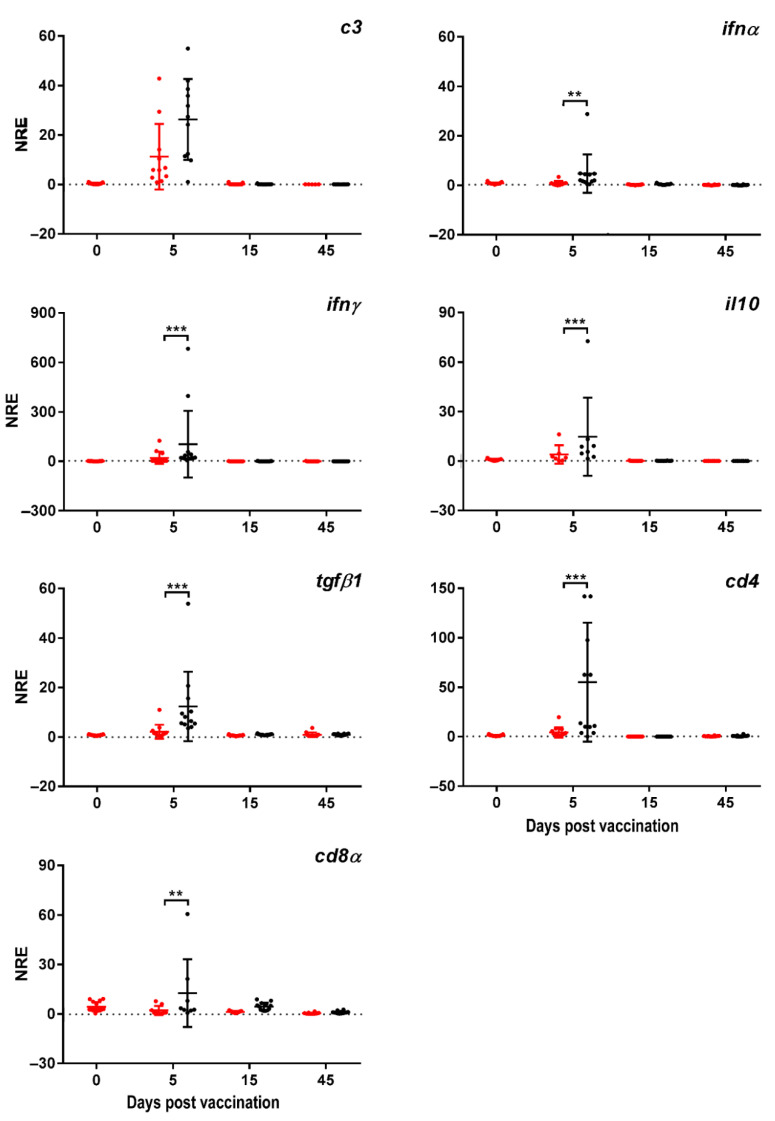
Normalized relative expression (NRE) of immune-related genes for vaccinated (black dots) and non-vaccinated Atlantic salmon (*Salmo salar*) head kidney. Fish reared under productive conditions were intraperitoneally vaccinated with ALPHA JECT LiVac^®^ SRS and sampled from both vaccinated and non-vaccinated fish at zero, 5-, 15-, and 45-days post-vaccination (dpv) (*n* = 15 fish per treatment and sampling time-point). Two-way ANOVA test and post-hoc Sidak’s multiple comparisons test was conducted (α = 0.05) to find differences in each time between control and vaccinated groups. Statistical differences were indicated when *p* value ≤ 0.05. Gene expression results are expressed as the mean ± SD. ** *p*-value < 0.01, *** *p*-value < 0.001.

At innate level, we observed a slight increase in the expression of *c3* at 5 dpv for the vaccinated group (26.3 ± 16.3) compared to the non-vaccinated (control) group (11.2 ± 13.2). However, this increase in the vaccinated group did not show significant difference compared to the non-vaccinated treatment (p > 0.05). No differences in expression were observed at 15 and 45 dpv between the experimental groups. By contrast, *ifnα* showed an augmentation in its expression at 5 dpv (4.74 ± 7.75) compared to the non-vaccinated group (0.86 ± 0.94). The expression of *ifnα* went back to basal values at 15 (0.43 ± 0.23, respectively) and 45 dpv (0.24± 0.12, respectively) (Figure 1). These data suggest that an interferon-mediated response could take place at 5 dpv in live-attenuated *P. salmonis*-vaccinated Atlantic salmon.

In order to determine whether the *ifnα* modulated the upregulation of other cellular-mediated immune response markers, we first evaluated the expression of *ifnγ*. Data registered the upregulation of *ifnγ* at 5 dpv in the vaccinated group (103.95 ± 202.6) compared to non-vaccinated fish (20.89 ± 36.98), reinforcing the hypothesis that an interferon-mediated cellular immune response could take place at early times post-vaccination in fish reared under an open sea regime. Then, the expression decreased to the control baseline value at 15 and 45 dpv (0.69 ± 0.46 and 0.29 ± 0.14 for vaccinated; 0.34 ± 0.20 and 0.40 ± 0.50, respectively) (Figure 1).

We also assessed the expression of *cd4* and *cd8α* in order to determine whether the upregulation of *ifnα* and *ifnγ* had an impact on these key immune cell membrane markers directly related to a cellular immunity context. In the case of *cd4*, there was a clear upregulation at 5 dpv in the vaccinated group (55.02 ± 60.08) compared to the non-vaccinated salmon (4.30 ± 5.01). The same effect at 5 dpv was observed for *cd8α* (12.62 ± 20.48 for vaccinated, and 2.14 ± 2.78 for non-vaccinated group, respectively). These data suggest that a T helper-mediated immune response might be promoted in the phenotype of response to the attenuated live SRS vaccine at early times post-treatment. The expression values for *cd4* and *cd8α* at 15 and 45 dpv for the vaccinated group (0.08 ± 0.02 and 4.47 ± 2.34; 0.73 ± 0.70 and 0.97 ± 0.74, respectively) dropped to the level of non-vaccinated fish (0.07 ± 0.06 and 1.24 ± 0.52; 0.55 ± 0.35 and 0.42 ± 0.44, respectively) (Figure 1).

At immune regulatory gene level, the expression of *il-10* and *tgfβ* was also upregulated at 5 dpv in the vaccinated group (14.75 ± 23.70 and 12.35 ± 14.00, respectively) compared to the non-vaccinated fish (3.96 ± 5.6 and 2.13 ± 2.82, respectively). In the same way as for most of the genes, the expression for *tgfβ* fell to the level of non-vaccinated fish both at 15 (0.97 ± 0.20) and 45 dpv (0.94 ± 0.31). No expression was reported for *il-10* at 15 and 45 dpv both in vaccinated and non-vaccinated fish (Figure 1).

Altogether, the results suggest the administration of the attenuated live SRS LiVac vaccine induces a transient short-term activation of the cellular-mediated immune response in the first days of vaccination, the upregulation peak being at 5 dpv.

## 4. Discussion

In this study, we evaluate the expression profile of a set of gene markers related to innate and adaptive immunity in the context of a cellular response in Atlantic salmon immunized with a live-attenuated vaccine against *P. salmonis*. We analyzed the expression at zero, 5-, 15- and 45-days post-vaccination reared under productive farm conditions. Our results reveal that the administration of the attenuated live SRS LiVac vaccine induces a short-term upregulation of the cellular-mediated immune response at 5 dpv modulated by the upregulation of *ifnα*, *ifnγ*, and the *cd4* and *cd8α* T cell surface markers.

Typical antimicrobial strategies used against *P. salmonis* include antibiotics and vaccines, but these applications have largely failed [13,27,28]. When an outbreak appears, the antibiotics are highly inefficient and abusive due to the intracellular nature of the infective cycle of *P. salmonis*. In fact, the amount of antibiotic that reaches the bacterium is minimal compared to the quantity released to the environment, which clearly represents a potential risk to human health and may also generate resistant bacteria [13,29,30].

Vaccination is considered one of the most effective strategies used to maintain human and animal health worldwide, but is a cost-effective method for controlling infectious diseases in aquaculture. From the development of the first vaccines for aquaculture, the use of environmentally unfriendly chemicals, especially antibiotics, have decreased in the industry. Vaccines used against *P. salmonis* have led to variable results, providing protection levels of less than 30% against bacterial outbreaks that occur immediately after transferring fish from fresh to saltwater. However, the fish become susceptible to a second and more aggressive outbreak of *P. salmonis*, due to a weakened immune response after the first immunization [13,29]. In general, most bacterin vaccines against SRS have not been fully protective and subunit vaccines appeared to be less effective in reducing total mortality or delaying the time of occurrence of the first outbreak of SRS. In 2016, the first attenuated-live vaccine against SRS was released to the market, called ALPHA JECT LiVac^®^ SRS vaccine from Pharmaq. However, up to now there is no data about the actors involved in the immune response induced under field conditions [15]. In this way, our study provides data about the effect of the vaccine in farm conditions.

The first developed vaccines were based on bacterins. The evaluation of this type of vaccine reflects that immunity induced is often less than for four months and mostly mediated by the humoral immune response, being an effective strategy for extracellular pathogens or pathogens producing toxins [31]. Recently, Meza et al. performed an evaluation of a *P.salmonis* inactivated vaccine, where the authors showed an increase of transcripts of secretory IgM (sIgM) after vaccination and challenge, together with a detectable titer of specific IgM in plasma, and a decreased expression of CD8*α* after challenge [32]. By contrast, live-attenuated vaccines retain the ability of the pathogen to infect the host and, consequently, the stimulation of the CD8+ T-cell response [31]. In this matter, the activation of an effective immune response by a live-attenuated vaccine against *P. salmonis* acquires particular relevance. In fact, *P. salmonis* is an intracellular pathogen able to survive and replicate inside of Atlantic salmon macrophages infected, evading the lysosomal response [23,33]. For this reason, it is very important that the vaccines used against intracellular pathogens succeed in inducing the cellular immune response in those infected cells [32,34,35,36]. In our study, we observed that the induction on the expression of molecules was associated with interferon-mediated immunity (*ifnα*; *ifnγ*). In the context of an intracellular infection, IFN-α is secreted during the innate response and is responsible of the subsequent IFN-stimulated genes expression. This mechanism is responsible for orchestrating the activation of the response against the intracellular pathogen infection [37]. Among those genes stimulated in response to IFNα, a key cytokine is involved in driving the response against intracellular pathogens. In fact, IFNγ is considered a key regulator for macrophage activation [38]. This is particularly important in the context of *P. salmonis* considering that infected macrophages exhibited fewer proteolytic foci and the induction of an anti-inflammatory environment that favors *P. salmonis* survival and replication [33,39]. Additionally, IFNγ is a cytokine that in higher vertebrates also promotes cell-mediated immunity, mainly through the activation of the Th1-type adaptive immune response [40]. In line with this concept, previous studies support the protective effect of cell-mediated immunity in the context of a Th1 response in salmonids [24,41,42]. In the adaptive cell-mediated immune response, IFNγ is produced by CD4+ T cells (Th1) and CD8+ T lymphocytes (cytotoxic T lymphocytes, CTL) [43], thus developing a pivotal role for the clearance of intracellular pathogens [44]. Accordingly, our study registered the coordinated upregulation of *ifnγ* and the immune cell surface markers *cd4*, and *cd8α* at the same time-point (5 dpv). In several fish species, the expression analysis of CD4 genes has suggested that teleost CD4+ cells may function as helper T cells, similar to mammalian CD4+ cells [45,46]. This evidence, together with the set of genes upregulated in our study, open the possibility that a Th1 response might take place in Atlantic salmon vaccinated with the called ALPHA JECT LiVac^®^ SRS vaccine. Importantly, the ability of live-attenuated vaccines to induce a Th1-response is poorly described and could even be pathogen-specific [36]. In fish, Shoemaker et al. showed that live vaccines stimulate a long-lasting cellular immunity [31]. This modulation was observed at 5 dpv, suggesting that the cellular-mediated immune response might be promoted in response to the attenuated live SRS vaccine only at early times post-treatment.

The promotion of an immune response activation in the first days post-vaccination observed in our study in not surprising. In teleost fish, injectable and oral vaccines only induce a short-term protection. This response might be explained in terms of antibody response, half-life, late affinity maturation, and immunological memory [21,47,48]. The different antibodies subpopulations have dissimilar kinetics, where the antibodies with a lower affinity are expressed early, transiently and with low titers. Antibodies with intermediate affinity appear not earlier than five weeks. Conversely, the antibodies with a higher affinity are expressed later in higher concentrations and persistently [49], which in channel catfish increased 100-fold, but only after 10 weeks post-immunization [50]. As in mammals, in teleost fish specific high-affinity antibodies produced by long-lived plasma cells (LLPCs) were recently described. A study reported the maximal number of LLPCs at the same time as the dynamics of serum antibody titers and affinity maturation [51]. Further studies are needed to determine the effect of the LiVac vaccine in this matter.

While many vaccines report CTL responses (mediated by CD8 regulation) for DNA vaccines or attenuated live virus preparations, many of them have limited efficiencies. The reasons behind this phenomenon remain unclear [52]. At least the importance of cell-mediated immunity against intracellular bacterial pathogens in fish has been demonstrated by Yamasaki et al. [53], where the authors compared the adaptive immune response and protection induced by live attenuated vaccine versus formalin-killed cells of *Edwardsiella tarda* in ginbuna crucian carp (*Carassius auratus langsdorfii*) challenged with *E. tarda*. As a result, the authors showed that after challenge with bacteria, live-attenuated vaccine induce high survival rates accomplished with a high IFNγ expression level and increased cytotoxic T lymphocytes (CTLs). In contrast, all fish vaccinated with bacterin died following *E. tarda* infection. Bacterin induced high IL-4/13A and IL-10 expression levels and increased antibody titers, whereas Th1-like response was suppressed [53,54]. In our results, together with the expression of molecules associated with the cellular immune response, an increase in the expression of *il-10* and *tgfβ* is observed, which, like the molecules associated with the cellular response, are only observed at 5 dpv, correlating with the short-term protection observed in injectable and oral vaccines used in aquaculture [21,47,48]. IL-10 is an anti-inflammatory cytokine and suppresses immune responses [55] through the regulation of pro-inflammatory cytokines [56]. On the other hand, TGFβ regulates the activation of a repertoire of immune cell populations and the promotion of tissue repair in the site where the local inflammatory response takes place [56,57]. Both cytokines have also shown a relevant role in the control of pathogenic infective processes of an intracellular nature [24,58]. The upregulation of *il-10* and *tgfβ*, together with the modulation of *ifnα, ifnγ,* and the cell surface markers *cd4 and cd8α*, suggest that a balanced activation of the immune response took place only at 5 dpv.

In conclusion, our results reveal that the administration of the attenuated live SRS LiVac vaccine induces a controlled short-term (only at 5-days post-vaccination) upregulation of the cellular-mediated immune response modulated by the upregulation of *ifnα*, *ifnγ*, the *cd4* and *cd8α* T cell surface markers, and the regulatory cytokines *il-10* and *tgfβ*. The scope of this short-term upregulation cellular-mediated immune response against *P. salmonis* natural outbreak in Atlantic salmon subjected to productive farm conditions deserves further research.

## Figures and Tables

**Table 1:** qRT-PCR condition details for the primers evaluated.

Gene Acronym	Activation Step	Number of Cycles	Denaturation Step	Annealing Step	Extension Step	Tm Product (°C)
*c3*	95 °C × 2 min	40	95 °C × 5 s	60 °C × 10 s	72 °C × 10 s	85.6
*ifnα*		40	95 °C × 5 s	60 °C × 10 s	72 °C × 10 s	83
*ifnγ*		40	95 °C × 5 s	60 °C × 10 s	72 °C × 10 s	81.8
*il-10*		40	95 °C × 5 s	60 °C × 10 s	72 °C × 10 s	82.5
*tgf-β*		40	95 °C × 5 s	60 °C × 10 s	72 °C × 10 s	80.1
*eIFα*		40	95 °C × 5 s	60 °C × 10 s	72 °C × 10 s	85.5
*18S*		40	95 °C × 5 s	60 °C × 10 s	72 °C × 10 s	82.7
*cd4*	95 °C × 2 min	40	95 °C × 5 s	57 °C × 10 s	72 °C × 10 s	83.8
*cd8a*	95 °C × 2 min	40	95 °C × 5 s	55 °C × 10 s	72 °C × 10 s	81.8

Tm: melting temperature.

## References

[1] FAO (2020). The State of World Fisheries and Aquaculture 2020.

[2] FAO (2019). Report of the FAO/MSU/WB First Multi-Stakeholder Consultation on a Progressive Management Pathway to Improve Aquaculture Biosecurity (PMP/AB). Proceedings of the Stakeholder Consultation on PMP for Improving Aquaculture Biosecurity.

[3] Tort L. (2011). Stress and immune modulation in fish. Dev. Comp. Immunol..

[4] Figueroa C., Veloso P., Espin L., Dixon B., Torrealba D., Elalfy I.S., Afonso J.M., Soto C., Conejeros P., Gallardo J.A. (2020). Host genetic variation explains reduced protection of commercial vaccines against Piscirickettsia salmonis in Atlantic salmon. Sci. Rep..

[5] Lafferty K.D., Harvell C.D., Conrad J.M., Friedman C.S., Kent M.L., Kuris A.M., Powell E.N., Rondeau D., Saksida S.M. (2015). Infectious diseases affect marine fisheries and aquaculture economics. Ann. Rev. Mar. Sci..

[6] Assefa A., Abunna F. (2018). Maintenance of Fish Health in Aquaculture: Review of Epidemiological Approaches for Prevention and Control of Infectious Disease of Fish. Vet. Med. Int..

[7] Brudeseth B.E., Wiulsrod R., Fredriksen B.N., Lindmo K., Lokling K.E., Bordevik M., Steine N., Klevan A., Gravningen K. (2013). Status and future perspectives of vaccines for industrialised fin-fish farming. Fish Shellfish Immunol..

[8] Aqua Sernapesca: “El SRS Sigue Siendo el Mayor Problema Sanitario que Enfrenta la Salmoniculttura”. http://www.aqua.cl/2012/11/23/sernapesca-el-srs-sigue-siendo-el-mayor-problema-sanitario-que-enfrenta-la-salmoniculttura/#.

[9] SERNAPESCA (2018). Informe Sanitario Acuícola año 2017.

[10] SERNAPESCA (2019). Informe Sanitario Acuícola año 2018.

[11] SERNAPESCA (2013). Informe Sanitario Acuícola año 2012.

[12] Marshall S.H., Conejeros P., Zahr M., Olivares J., Gomez F., Cataldo P., Henriquez V. (2007). Immunological characterization of a bacterial protein isolated from salmonid fish naturally infected with Piscirickettsia salmonis. Vaccine.

[13] Maisey K., Montero R., Christodoulides M. (2017). Vaccines for piscirickettsiosis (salmonid rickettsial septicaemia, SRS): The Chile perspective. Expert Rev. Vaccines.

[14] SERNAPESCA (2019). Informe Sobre uso de Antimicrobianos en la Salmonicultura Nacional.

[15] Flores-Kossack C., Montero R., Köllner B., Maisey K. (2020). Chilean aquaculture and the new challenges: Pathogens, immune response, vaccination and fish diversification. Fish Shellfish Immunol..

[16] Rozas M., Enriquez R. (2014). Piscirickettsiosis and Piscirickettsia salmonis in fish: a review. J Fish Dis.

[17] Evensen O. (2016). Immunization Strategies against Piscirickettsia salmonis Infections: Review of Vaccination Approaches and Modalities and Their Associated Immune Response Profiles. Front. Immunol..

[18] Sunyer J.O. (2013). Fishing for mammalian paradigms in the teleost immune system. Nat. Immunol..

[19] Uribe C., Folch H., Enriquez R., Moran G. (2011). Innate and adaptive immunity in teleost fish: A review. Vet. Med..

[20] Alvarez-Pellitero P. (2008). Fish immunity and parasite infections: From innate immunity to immunoprophylactic prospects. Vet. Immunol. Immunopathol..

[21] Tobar I., Arancibia S., Torres C., Vera V., Soto P., Carrasco C., Alvarado M., Neira E., Arcos S., Tobar J.A. (2015). Successive Oral Immunizations Against Piscirickettsia Salmonis and Infectious Salmon Anemia Virus are Required to Maintain a Long-Term Protection in Farmed Salmonids. Front. Immunol..

[22] Inohara N., Koseki T., Chen S., Wu X., Nunez G. (1998). CIDE, a novel family of cell death activators with homology to the 45 kDa subunit of the DNA fragmentation factor. EMBO J..

[23] Pérez-Stuardo D., Espinoza A., Tapia S., Morales-Reyes J., Barrientos C., Vallejos-Vidal E., Sandino A.M., Spencer E., Toro-Ascuy D., Rivas-Pardo J.A. (2020). Non-Specific Antibodies Induce Lysosomal Activation in Atlantic Salmon Macrophages Infected by Piscirickettsia salmonis. Front. Immunol..

[24] Reyes-López F.E., Romeo J.S., Vallejos-Vidal E., Reyes-Cerpa S., Sandino A.M., Tort L., Mackenzie S., Imarai M. (2015). Differential immune gene expression profiles in susceptible and resistant full-sibling families of Atlantic salmon (*Salmo salar*) challenged with infectious pancreatic necrosis virus (IPNV). Dev. Comp. Immunol..

[25] Peña A.A., Bols N.C., Marshall S.H. (2010). An evaluation of potential reference genes for stability of expression in two salmonid cell lines after infection with either Piscirickettsia salmonis or IPNV. BMC Res. Notes.

[26] Livak K.J., Schmittgen T.D. (2001). Analysis of relative gene expression data using real-time quantitative PCR and the 2-ÄÄCT method. Methods.

[27] Bravo S., Midtlyng P. (2007). The use of fish vaccines in the Chilean salmon industry 1999–2003. Aquaculture.

[28] Tobar J.A., Jerez S., Caruffo M., Bravo C., Contreras F., Bucarey S.A., Harel M. (2011). Oral vaccination of Atlantic salmon (*Salmo salar*) against salmonid rickettsial septicaemia. Vaccine.

[29] Miranda C.D., Godoy F.A., Lee M.R. (2018). Current Status of the Use of Antibiotics and the Antimicrobial Resistance in the Chilean Salmon Farms. Front. Microbiol..

[30] Millanao B.A., Barrientos H.M., Gómez C.C., Tomova A., Buschmann A., Dölz H., Cabello F.C. (2011). Injudicious and excessive use of antibiotics: Public health and salmon aquaculture in Chile. Rev. Med. Chile.

[31] Shoemaker C.A., Klesius P.H., Evans J.J., Arias C.R. (2009). Use of modified live vaccines in aquaculture. J. World Aquac. Soc..

[32] Meza K., Inami M., Dalum A.S., Lund H., Bjelland A.M., Sorum H., Lovoll M. (2019). Comparative evaluation of experimental challenge by intraperitoneal injection and cohabitation of Atlantic salmon (*Salmo salar* L.) after vaccination against Piscirickettsia salmonis (EM90-like). J. Fish Dis..

[33] Pérez-Stuardo D., Morales-Reyes J., Tapia S., Ahumada D.E., Espinoza A., Soto-Herrera V., Brianson B., Ibaceta V., Sandino A.M., Spencer E. (2019). Non-lysosomal Activation in Macrophages of Atlantic Salmon (*Salmo salar*) After Infection With Piscirickettsia salmonis. Front. Immunol..

[34] Beck B.H., Peatman E. (2015). Mucosal Health in Aquaculture.

[35] Rozas-Serri M., Pena A., Arriagada G., Enriquez R., Maldonado L. (2018). Comparison of gene expression in post-smolt Atlantic salmon challenged by LF-89-like and EM-90-like Piscirickettsia salmonis isolates reveals differences in the immune response associated with pathogenicity. J. Fish Dis..

[36] Seder R.A., Hill A. (2000). V Vaccines against intracellular infections requiring cellular immunity. Nature.

[37] Gan Z., Chen S.N., Huang B., Zou J., Nie P. (2020). Fish type I and type II interferons: Composition, receptor usage, production and function. Rev. Aquac..

[38] Green D.S., Young H.A., Valencia J.C. (2017). Current prospects of type II interferon γ signaling & autoimmunity. J. Biol. Chem..

[39] Álvarez C.A., Gomez F.A., Mercado L., Ramírez R., Marshall S.H. (2016). Piscirickettsia salmonis imbalances the innate immune response to succeed in a productive infection in a salmonid cell line model. PLoS ONE.

[40] Constant S.L., Bottomly K. (1997). Induction of Th1 and Th2 CD4+ T cell responses: The alternative approaches. Annu. Rev. Immunol..

[41] Sun B., Skjæveland I., Svingerud T., Zou J., Jørgensen J., Robertsen B. (2011). Antiviral activity of salmonid gamma interferon against infectious pancreatic necrosis virus and salmonid alphavirus and its dependency on type I interferon. J. Virol..

[42] Xu C., Guo T.-C., Mutoloki S., Haugland O., Evensen O. (2012). Gene expression studies of host response to Salmonid alphavirus subtype 3 experimental infections in Atlantic salmon. Vet. Res..

[43] Robertsen B. (2006). The interferon system of teleost fish. Fish Shellfish Immunol..

[44] Boehm U., Klamp T., Groot M., Howard J.C. (1997). Cellular responses to interferon-gamma. Annu. Rev. Immunol..

[45] Maisey K., Montero R., Corripio-Miyar Y., Toro-Ascuy D., Valenzuela B., Reyes-Cerpa S., Sandino A.M., Zou J., Wang T., Secombes C.J. (2016). Isolation and Characterization of Salmonid CD4+ T Cells. J. Immunol..

[46] Takizawa F., Magadan S., Parra D., Xu Z., Koryta T., Boudinot P., Sunyer J.O. (2016). Novel Teleost CD4-Bearing Cell Populations Provide Insights into the Evolutionary Origins and Primordial Roles of CD4+ Lymphocytes and CD4+ Macrophages. J. Immunol..

[47] Ye J., Bromage E.S., Kaattari S.L. (2010). The strength of B cell interaction with antigen determines the degree of IgM polymerization. J. Immunol..

[48] Kaattari S.L., Zhang H.L., Khor I.W., Kaattari I.M., Shapiro D.A. (2002). Affinity maturation in trout: Clonal dominance of high affinity antibodies late in the immune response. Dev. Comp. Immunol..

[49] Ye J., Kaattari I.M., Kaattari S.L. (2011). The differential dynamics of antibody subpopulation expression during affinity maturation in a teleost. Fish Shellfish Immunol..

[50] Wu L., Fu S., Yin X., Leng W., Guo Z., Wang A., Ye J. (2019). Affinity maturation occurs in channel catfish (*Ictalurus punctaus*) following immunization with a T-cell dependent antigen. Fish Shellfish Immunol.

[51] Wu L., Fu S., Yin X., Guo Z., Wang A., Ye J. (2019). Long-Lived Plasma Cells Secrete High-Affinity Antibodies Responding to a T-Dependent Immunization in a Teleost Fish. Front. Immunol..

[52] Yamaguchi T., Takizawa F., Furihata M., Soto-Lampe V., Dijkstra J.M., Fischer U. (2019). Teleost cytotoxic T cells. Fish Shellfish Immunol..

[53] Yamasaki M., Araki K., Maruyoshi K., Matsumoto M., Nakayasu C., Moritomo T., Nakanishi T., Yamamoto A. (2015). Comparative analysis of adaptive immune response after vaccine trials using live attenuated and formalin-killed cells of Edwardsiella tarda in ginbuna crucian carp (Carassius auratus langsdorfii). Fish Shellfish Immunol..

[54] Nakanishi T., Shibasaki Y., Matsuura Y. (2015). T Cells in Fish. Biology.

[55] Zou J., Secombes C.J. (2016). The function of fish cytokines. Biology.

[56] Reyes-Cerpa S., Maisey K., Reyes-Lpez F., Toro-Ascuy D., Mara A., Imarai M. (2013). Fish Cytokines and Immune Response. New Advances and Contributions to Fish Biology.

[57] Li F., Zeng B., Chai Y., Cai P., Fan C., Cheng T. (2009). The linker region of Smad2 mediates TGF- b -dependent ERK2-induced collagen synthesis. Biochem. Biophys. Res. Commun..

[58] Reyes-Cerpa S., Reyes-López F., Toro-Ascuy D., Montero R., Maisey K., Acuña-Castillo C., Sunyer J.O., Parra D., Sandino A.M., Imarai M. (2014). Induction of anti-inflammatory cytokine expression by IPNV in persistent infection. Fish Shellfish Immunol..

